# Secondary amenorrhoea due to pheochromocytoma: a case report

**DOI:** 10.1186/1757-1626-1-30

**Published:** 2008-07-11

**Authors:** Ammar Wakil, Stephen Atkin

**Affiliations:** 1 Michael White Diabetes Centre, Hull Royal Infirmary 220-236 Anlaby Road, Hull, HU3 2RW, UK

## Abstract

**Introduction:**

Pheochromocytoma is a chromaffin cell catecholamine-secreting tumour originating from the adrenal medulla.

**Case presentation:**

We report here an unusual presentation of pheochromocytoma with secondary amenorrhoea and its resolution after medical treatment with the alpha adrenergic blocker, Phenoxybenzamine.

**Conclusion:**

Pheochromocytoma is a rare cause of secondary amenorrhoea.

## Introduction

Pheochromocytomas are chromaffin cell catecholamine-secreting tumours originating from the adrenal medulla. They may present in a number of ways from an incidental finding to that of the classical triad of episodic headache, tachycardia and sweating due to hypersecretion of catecholamines resulting in episodic hypertension[1]. However, secondary amenorrhea, potentially through activation of alpha receptors, has not been reported as a presentation.

## Case presentation

A 35 year white Caucasian nulliparous female presented to the fertility clinic with 18 months history of secondary amenorrhoea that was ascribed to post pill amenorrhoea. She had a regular menstrual cycle since the age of 15. At the age of 18 she started taking the contraceptive pill and she had a regular withdrawal bleeding every 28 days, but amenorrhoea ensued following its cessation at the age of 33. There was also no history of eating disorder or recent weight loss, no previous medical history and there was no family history of relevance including autoimmune disease. She was a non smoker, who drank less than 10 units of alcohol a week. Physical examination revealed a lady with normal blood pressure of 128/78 mmHg, weight of 60 kg, height of 173 cm giving her a Body Mass Index of 20 kg/m^2^.

Her investigations in the fertility clinic revealed iron deficiency anaemia, Hb 9.7 g/dl, microcytosis and a low ferritin of 4 ug/L (15–30 Units). She had luteal phase FSH, LH, oestradiol and progesterone of 7.9 iu/L (0–5), 3.0 i.u/L (2–9), <70 pmol/L (150–1000) and 1 nmol/L, respectively. Her prolactin was 220 mU/L (0–550) and testosterone was 1.3 nmol/L (0–4.1) while the free androgen index was 3 (0–8) and SHBG was 42 nmol/L. Her liver enzymes, electrolytes, urea and creatinine were all within the normal range. Her ultrasound of the pelvis revealed normal ovaries and normal endometrial thickness and hysterosalpingography showed patent fallopian tubes. Her iron deficiency anaemia was corrected by ferrous sulphate treatment. Induction of ovulation with clomiphene 150 mg for 5 days and two trials with gonadotrophins neither achieved pregnancy nor ovulation, with a complete failure of oocytes response. Meanwhile, she underwent investigations for the initial iron deficiency anaemia with gastroscopy and colonoscopy CT. The latter revealed an incidental left adrenal mass of 3 cm in diameter that did not show features typical of an adenoma. Two urinary 24 hour urinary fractionated catecholamines showed an excess secretion of norepinephrine at 1567 and 1159 nmol/L (NR 70–550) with normal epinephrine and dopamine secretion. She was treated initially with an escalating dose of phenoxybenzamine starting at 10 mg twice daily before adding propranolol slow release 80 mg daily prior to undergoing surgery. Two weeks after starting the phenoxybenzamine, she reported resumption of regular menstrual periods after 24 months of secondary amenorrhoea. Definitive treatment in the form of laparoscopic left adrenalectomy confirmed the presence of an encapsulated pheochromocytoma that was completely excised.

## Discussion

This unusual presentation of pheochromocytoma has not been reported before. One possibility is that ovarian granulsa luteal cells in the human and animal have alpha and beta adrenergic receptors. It was shown recently that activation of alpha adrenergic receptors causes inhibition of LH and HCG mediated progesterone release[2] through intracellular Ca^++ ^and phosphoinositide pathways[2] although at least in rats, the renin angiotensin system is thought to play a role in mediating the effect of norepinephrine [3] in the ovary. In the group given Doxazocin, there was a significant increase in serum progesterone and ovarian renin angiotensin system soluble and membrane-bound enzymes confirming that alpha adrenergic receptors caused inhibition of progesterone release.

A less likely possibility is the interaction of norepinephrine at the level of the hypothalamus. There is animal evidence that GnRH release and LH pulsatilty are modulated by extrahypothalamic norepinephrine [4]. The median pre-optic area of the hypothalamus is innervated by ascending noradrenergic fibres from the locus coeruleus and these fibres stimulate GnRH and LH release [4]. Animal studies have shown that alpha adrenergic receptors in the hypothalamus play a role in modulating GnRH and LH release. In a study on rats, stimulating the ascending noradrenergic bundle has resulted in partial or complete inhibition of the LH pulse pattern[5]. The mechanism of this effect remains unknown[4]. However, this was not found in human studies. As an example, intravenous administration of the alpha adrenergic-blocking agent, phentolamine, in amounts sufficient to produce marked orthostatic hypotension in five normal men, resulted in no alteration of LH secretory spike activity[6]. In our patient, the use of GnRH for induction of ovulation was unsuccessful which suggests that the level of interference by the excess tumorous norepinephrine was at the ovary rather than the hypothalamus.

## Conclusion

This case report illustrates a patient presented with amenorrhoea that was secondary to a pheochromocytoma, a rare differential diagnosis for patients presenting with secondary amenorrhoea.

## Abbreviations

Hb: haemoglobin; FSH: follicular stimulating hormone; SHBG: sex hormone binding globulin; LH: leutinizing hormone; NR: normal range; HCG: human chorionic gonadotropin; GnRH: gonadotropin releasing hormone; C^++^: ionized calcium.

## Competing interests

The authors declare that they have no competing interests.

## Authors' contributions

SLA conceived the study, coordination and helped to draft the manuscript. AW reviewed the clinical records, wrote and submitted the case for publication. All authors read and approved the final manuscript.

## Consent

Written informed consent was obtained from the patient. A copy of the written consent is available for review by the Editor-in-Chief of this journal.
